# Editorial: Immunity to *Neisseria gonorrhoeae*

**DOI:** 10.3389/fimmu.2020.01375

**Published:** 2020-07-02

**Authors:** Michael W. Russell, Scott D. Gray-Owen, Ann E. Jerse

**Affiliations:** ^1^Department of Microbiology and Immunology, University at Buffalo, Buffalo, NY, United States; ^2^Department of Molecular Genetics, University of Toronto, Toronto, ON, Canada; ^3^Department of Microbiology and Immunology, F. Edward Hebert School of Medicine, Uniformed Services University, Bethesda, MD, United States

**Keywords:** *Neisseria gonorrhoeae*, immunity, vaccine, immune evasion, immunopathogenesis

Immunity to *Neisseria gonorrhoeae* has been hard to define because, until recently, there has been no evident state of immunity to gonorrhea in humans; re-infections are fairly common, implying that an episode of infection does not usually induce a protective immune response. Experimental investigation is complicated by the fact that humans are the only known natural host for this infection. However, recent experimental findings using a variety of *in vitro* and *in vivo* model systems, in addition to clinical and epidemiological studies, have begun to cast new light on this problem. This Research Topic brings together 12 papers from leading contributors to the field, affording a comprehensive overview of current understanding and suggesting future pathways of investigation that should lead to actionable understanding of the immuno-pathogenesis of gonococcal infection, as well as facilitate novel approaches to therapeutic treatment and the development of a prophylactic vaccine. The need for such innovations is highlighted by the ongoing emergence of resistance in *N. gonorrhoeae* to all currently available antibiotics, which threatens to render gonorrhea untreatable (1), as well as the unacceptably high prevalence of gonorrhea. Global incidence is now estimated at 87 million new infections every year (2), with the heaviest burden of reproductive tract morbidity falling on women, especially in low- and middle-income countries and underserved populations.

The articles in this Research Topic fall broadly into two connected threads: human immune responses to gonococcal infection; and gonococcal vaccine development. First, Lovett and Duncan set the scene with a discussion of what is currently known about the human immune response to *N. gonorrhoeae* and the natural history of the infection. Crucially, many cervical and pharyngeal infections are “asymptomatic,” meaning that the subject is unaware of her or his infection. This vitiates the evaluation of host responses that typically develop with the onset of inflammation and profuse exudation of neutrophils. Further complications in studying immune responses arise because of the antigenic diversity of *N. gonorrhoeae* as well as similarity to commensal *Neisseria* species that frequently occur in the oropharynx. Antibody responses remain weak until upper tract infection ensues, but, by that stage, inflammatory damage develops with potentially serious consequences such as scarring of the epididymis or Fallopian tubes, which can lead to infertility in both sexes, and ectopic pregnancy and pelvic inflammatory disease in women. The pathogenesis of upper tract infection and the associated host responses are reviewed by Lenz and Dillard, focusing especially on the use of human tissue explants, which complements findings derived from animal models in which infection is unnatural.

Gonococcal infection enhances the transmission and acquisition of HIV, and the mechanisms of this poorly understood interaction are discussed by Guvenc et al., revealing multifactorial, bidirectional effects. Mechanisms include the ability of *N. gonorrhoeae* to selectively induce Th17 responses and concomitantly suppress Th1/2-driven adaptive immune responses through several pathways, compromising immune functionality and increasing the primary target Th17 cells for HIV. In addition, the NFκB-dependent pro-inflammatory effects of several gonococcal components can promote HIV transcription, while gonococcal interactions with different mucosal antigen-presenting cells also appear to either enhance or oppose viral replication and immunity. The prevalence of *N. gonorrhoeae* means that these interactions have significant impact on the ongoing HIV pandemic.

The profuse exudation of neutrophils into the male and female genital tracts characterizes symptomatic infection and constitutes a classical diagnostic criterion, yet apparently intact gonococci are frequently observed within these phagocytes on microscopic examination of exudate smears. Several mechanisms have been elucidated whereby *N. gonorrhoeae* resists intracellular destruction within neutrophils (3), and here Handing et al. demonstrate the role of the MtrCDE efflux pump, which has previously been recognized to be important in the resistance of *N. gonorrhoeae* to host antimicrobial peptides and antibiotics (4, 5), in avoidance of neutrophil killing. While neutrophils are the prime responders during gonorrhea, the singular focus on neutrophils has detracted from considering the role of other “professional phagocytes,” i.e., macrophages, which typically follow the influx of neutrophils in pyogenic infections. Although the presence of macrophages has been observed in the female mouse model of gonococcal infection (6), few studies have addressed their role. The review by Escobar et al. draws attention to the ability of *N. gonorrhoeae* to modulate macrophage differentiation into the “alternative” M2 pathway, and the consequences of this for gonococcal survival. Therapeutic strategies aimed at reversing this macrophage polarization, such as by use of COX-2 inhibitors, might have a beneficial effect.

Nutritional immunity, defined as the prevention of infection through deprivation of essential nutrients, is well-exemplified by the with-holding of metals such as iron and zinc by means of high-affinity chelating proteins. Pathogenic Neisseria express several proteins to acquire these metals from the host, the best known being the transferrin- and lactoferrin-binding proteins. Yadav et al. review the molecular structures of these and other metal-acquiring proteins, thereby illustrating their mechanisms of action.

Interest in developing vaccines against *N. gonorrhoeae* has been reinvigorated by the emergence of multidrug resistance, as well as the recent findings concerning the immuno-pathogenesis of gonococcal infection and the response to it, as reviewed by Russell et al.. A landmark paper by Petousis-Harris et al. (7) reported that recipients of a meningococcal vaccine (MeNZB), based on outer membrane vesicles (OMV) from a group B strain of *N. meningitidis*, were 31% less likely to be diagnosed later with gonorrhea than unimmunized subjects. This represented the first report of a state of protective immunity against gonorrhea in humans. Petousis-Harris and Radcliff review this and subsequent studies, and discuss potential mechanisms underlying cross-protection, including the possible induction of antibodies against shared antigens. OMV have the advantage of combining multiple individual antigens, including proteins as well as lipo-oligosaccharide, in one vaccine, which might explain the cross-protection of MeNZB against gonorrhea in humans, as well as the effectiveness of an experimental gonococcal OMV vaccine against antigenically diverse strains of *N. gonorrhoeae* in mice (8). However, numerous defined antigens have also been proposed as potential vaccine candidates [(9, 10), Russell et al.]. Reverse vaccinology has been successful in identifying several proteins for inclusion in the recently licensed meningococcal vaccine, Bexsero® (marketed by GlaxoSmithKline), and the use of this approach combined with immuno-proteomics and bioinformatics studies to identify novel gonococcal antigens is reviewed by Baarda et al.. More classical microbiological approaches have led to promising efforts to target a lipo-oligosaccharide epitope, designated 2C7, which is present in >95% of *N. gonorrhoeae* strains. Gonococcal lipo-oligosaccharide itself is highly reactogenic and contains numerous other epitopes that are variably expressed by different isolates, but an alternative approach focusing on 2C7 has been developed by means of peptide mimics, as discussed by Gulati et al.. Because expression of transferrin-binding proteins is required for human (male) infection by *N. gonorrhoeae* (11), they have been proposed as potential vaccine antigens. To overcome limitations imposed by their variable antigenic structure and other factors, Fegan et al. have created hybrids of transferrin-binding proteins A and B to use as vaccine immunogens capable of inducing bactericidal antibodies. Finally, Jen et al. describe gonococcal methionine sulfoxide reductase, which is required for gonococcal resistance to oxidative stress, as a novel vaccine candidate.

Thus, the stage is now set for significant advances in comprehending immunity to *N. gonorrhoeae* and in developing an effective vaccine against gonorrhea. The contributions to this Research Topic reveal a remarkable range of experimental models and technologies that have been brought to bear upon solving these problems. What is now required is translation of experimental findings to the human disease, with the objective of delivering novel interventions for the treatment and prevention of gonococcal disease and its devastating sequelae.

## Author Contributions

MR wrote the first draft of this editorial. All authors reviewed and edited the final document.

## Conflict of Interest

MR serves as a paid consultant for Therapyx, Inc., which is developing sustained release microparticulate adjuvants for use in inflammatory disease therapy and gonococcal vaccine development. SG-O is co-founder of Engineered Antigens Inc., which is focused on protein structure-based design of vaccine immunogens targeting pathogens including *N. gonorrhoeae*. The remaining author declares that the research was conducted in the absence of any commercial or financial relationships that could be construed as a potential conflict of interest.
